# Robot-Assisted Partial Nephrectomy with a New Robotic Surgical System: Feasibility and Perioperative Outcomes

**DOI:** 10.1089/end.2022.0140

**Published:** 2022-11-03

**Authors:** Weifeng Xu, Jie Dong, Yi Xie, Guanghua Liu, Jingmin Zhou, Huizhen Wang, Shengjie Zhang, Hui Wang, Zhigang Ji, Liang Cui

**Affiliations:** ^1^ Department of Urology, Peking Union Medical College, Chinese Academy of Medical Sciences, Peking Union Medical College Hospital, Beijing, China.; ^2^ Department of Operation Room, Peking Union Medical College, Chinese Academy of Medical Sciences, Peking Union Medical College Hospital, Beijing, China.; ^3^ Department of Urology, Civil Aviation General Hospital, Civil Aviation Medical College of Peking University, Beijing, China.

**Keywords:** nephrectomy, robotic surgical procedures, partial nephrectomy, laparoscopy

## Abstract

**Purpose:**

The aim of this study was to evaluate the feasibility and safety of a novel robotic system (KD-SR-01) for partial nephrectomy.

**Methods:**

Seventeen patients with small renal masses (SRMs) (≤4 cm) underwent KD-SR-01 robotic partial nephrectomy (KD-RPN) from December 2020 to March 2021 in our institution. The operative outcomes and perioperative data, including clinical and histological data, were prospectively collected and analyzed.

**Results:**

In total, 10 men and 7 women, with a median age of 51 years, underwent KD-RPN. Four transperitoneal procedures and 13 retroperitoneal procedures were performed without conversion to open or conventional laparoscopic surgery. The docking time and robotic operative time were 3.3 and 68.6 minutes, respectively. The warm ischemia time was 16.9 minutes. No major intraoperative or postoperative complications (Clavien grade ≥III) occurred. The duration of postoperative hospital stay was 5 days. Pathologic examination revealed nine clear cell carcinoma, two papillary cell carcinoma, one oncocytoma, and five angiomyolipoma cases. All surgical margins were negative. The estimated glomerular filtration rate (eGFR) on the first postoperative day was significantly decreased compared with the preoperative eGFR (91.7 ± 12.9 mL/min *vs* 97.9 ± 10.7 mL/min, *p* = 0.036). However, no significant difference was observed between the preoperative eGFR and the value on the fourth postoperative day (95.7 ± 13.4 mL/min *vs* 97.9 ± 10.7 mL/min, *p* = 0.427).

**Conclusions:**

KD-RPN was safe and feasible for treatment of SRM. The early oncologic and functional outcomes were promising. Long-term follow-up and well-designed prospective comparative studies with the da Vinci^®^ platform are needed to corroborate these findings.

## Introduction

The incidence of renal-cell carcinoma has increased worldwide in recent years due to the widespread use of imaging techniques to detect renal masses.^1,2^ Partial nephrectomy (PN) has distinguished itself from radical nephrectomy (RN) by demonstrating both equal oncological control and better renal functional outcomes, which have made PN the new standard surgical approach for T1a renal tumors.^3^

Laparoscopic partial nephrectomy (LPN) affords functional outcomes, oncologic outcomes, and complication rates equivalent to those of open partial nephrectomy and has become one of the standard treatments for small renal masses (SRMs).^4^ However, the laparoscopic technique remains difficult for the average urologist because of the technical challenges of performing tumor resection and renal reconstruction within limited time.^5^ The introduction of robotic technology allows most surgeons without experience to perform complex procedures more easily than the conventional laparoscopic approach.

Robotic surgery has demonstrated some technical advantages, such as magnified visualization, three-dimensional (3D) visualization, full articulation of instruments under precise control, absence of the fulcrum effect, and elimination of tremors.^6^ These technical advances decrease the technical difficulty associated with the critical portion of PN, including tumor dissection and pelvicaliceal renal reconstruction.^7^

The US Food and Drug Administration (FDA) has approved five robotic systems to date: AESOP, EndoAssist, Neuromate, Zeus, and da Vinci^®^.^8^ However, the da Vinci surgical system (Intuitive Surgical) has become nearly synonymous with the term “robotic surgery” due to its superior products, intellectual property protection, and worldwide training centers. da Vinci systems have been introduced worldwide over the last 20 years and are mainly used in urological, gynecological, and visceral surgeries.^8,9^ Nevertheless, implementation of the da Vinci platform is limited by the high cost of the systems.

In recent years, several devices have been under development in Europe and Asia^10–12^ and may stimulate a new era of robotic master–slave systems. In China, a new robotic platform called KD-SR-01™ (SuZhou Kang Duo Robot Co., Ltd., Suzhou, China), which includes an open surgical console, three robotic arms, and a 3D video imaging system, has been developed. The KD-SR-01 robotic system completed PN in animal experiments. Compared with traditional 3D laparoscopic surgery, KD-SR-01 robotic partial nephrectomy (KD-RPN) showed no significant difference in operation time or other parameters, but the KD-SR-01 robotic system has significant ergonomic advantages.^13^

The aim of this prospective single-institution study was to evaluate the feasibility and safety of the KD-SR-01 robotic system in a consecutive series of patients undergoing PN.

## Materials and Methods

### Patients

This prospective single-institution study included patients who underwent KD-RPN at the Department of Urology at Peking Union Medical College Hospital (PUMCH), Beijing, China, between December 2020 and March 2021. Our institutional review board approved the study. All patients were informed about the surgical technique and signed a written informed consent form that presented the risk of standard laparoscopic and/or laparotomic conversion. Clinical data of the study population were anonymously collected in an electronic database at the time of recruitment.

The inclusion criteria were as follows: (1) 18–75 years old, regardless of sex; (2) indications for PN; (3) TNM tumor stage of T1a; and (4) R.E.N.A.L. score ≤9.^14^ The exclusion criteria were as follows: (1) patients with severe uncontrolled infection; (2) patients with recurrent tumors; (3) patients with a previous upper abdominal surgical history; (4) patients with cardiovascular and cerebrovascular diseases, blood system diseases, and immune system diseases or diabetes, who could not meet the requirements to undergo surgery; and (5) pregnant or lactating women.

### Surgical team

Before starting the robotic PN with the KD-SR-01 system, our team consisting of two urologists and two nurses received training for 2 days in a dry laboratory with a third day in a wet laboratory, performing various surgical procedures with this system on pigs. All of the procedures included in this article were performed by members of this highly trained team. In addition, this surgical team has performed more than 500 cases of various operations with the da Vinci system.

### KD-SR-01 system

**FIG. 1.**
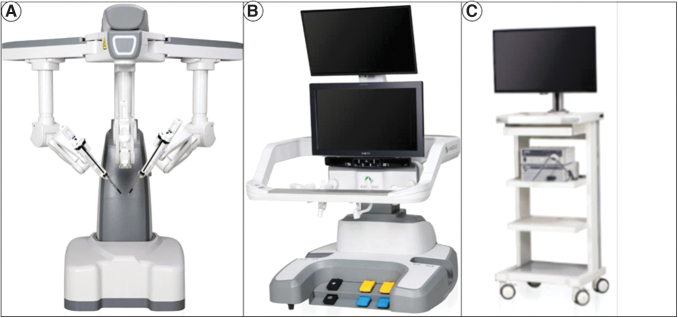
KD-SR-01 robotic system. **(A)** Patient cart, including three robotic arms; **(B)** surgical console; and **(C)** 3D imaging system.

**FIG. 2.**
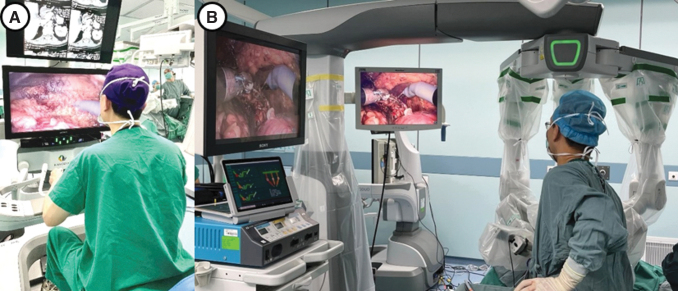
Intraoperative scene of KD-SR-01-assisted PN. **(A)** The surgeon sits in front of the open console to operate. **(B)** The positions of the assistant, robotic arms, and monitoring system. PN = partial nephrectomy.

The KD-SR-01 system consists of a surgical console, three robotic arms, and a 3D imaging system (Fig. 1A–C). The open console, integrating two master manipulators and a 3D high-definition monitor, enabled surgeons to precisely and synchronously control the surgical arms and instruments with passive polarized glasses without flexion of the neck (Fig. 2A).^13^

Unlike the four-arm system of the da Vinci robotic platform, the patient bed of the KD-SR-01 platform includes a three-arm system (Fig. 2B). The surgical instrument has 7° of freedom of movement and can filter out tremors of the hands. The imaging system adopts a modular interface, which can match the 3D laparoscopic display system of various brands that have been listed. In this study, Karl Storz IMAGE1 SD3-Link^®^ laparoscopic systems were used with 30°, 10-mm, 3D video laparoscopes.

KD-SR-01 will be approved for registration (National Medical Products Administration, NMPA) in China in the near future. At present, this research project with KD-SR-01 has passed the inspection of medical devices by the China NMPA and obtained the test report. In 2019, KD-SR-01 passed the NMPA special channel for innovative medical devices and was allowed to enter clinical trial research by NMPA.

This study was approved by the ethics committee of our institution.

### Surgical technique

Both retroperitoneal and transperitoneal approaches were administered in our series. The choice of surgical approach was based on the patient and tumor characteristics and surgeon's experience. The retroperitoneal approach was performed in most tumors, including posterior tumors, lateral tumors, and a part of anterior tumors. The transperitoneal approach was performed only in tumors on the medial side of the kidney and a part of anterior tumors close to the renal hilum.

#### Retroperitoneal approach

**FIG. 3.**
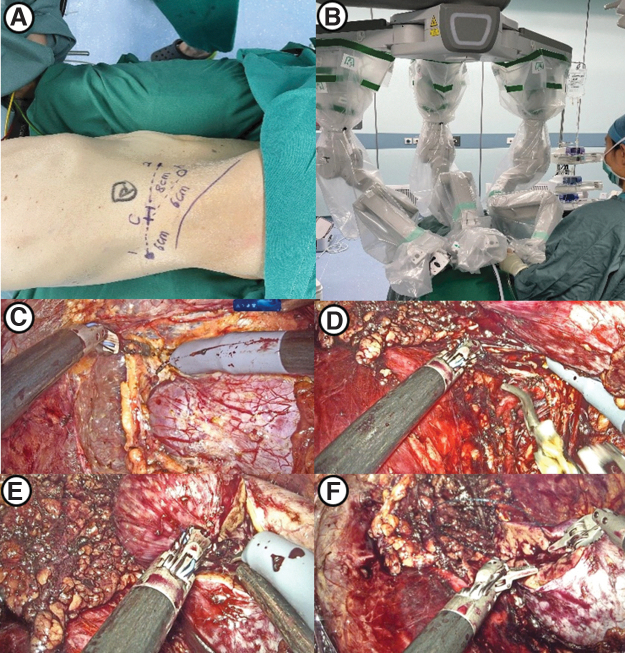
Surgical procedures of KD-SR-01-assisted PN using the retroperitoneal approach. **(A)** Template of port placement using the retroperitoneal approach (sites 1 and 2 for robotic instruments, site C for the camera, and site A for the assistant port). **(B)** Distribution of robotic arms. **(C)** Free kidney and exposed renal tumor. **(D)** Dissection and clamping of the renal artery. **(E)** Resection of the renal tumor. **(F)** Reconstruction of the renal parenchyma using continuous sutures.

After general anesthesia induction, the patient was placed in a lateral flank position. First, a 2-cm incision was made at the midaxillary line 2 cm above the iliac crest, through which the retroperitoneal cavity was created by balloon dilation. After a pneumoperitoneum of 14 mm Hg was established, a 30° 3D video laparoscope was introduced through the first incision. Then, two 10-mm robotic cannulas were placed under direct vision at the anterior and posterior axillary lines ∼2–3 cm above the iliac crest. Another 12-mm trocar was placed for assistance between the camera port and the anterior port (Fig. 3A, B).

After docking was performed, the standard technique was used to expose the kidney. The renal artery was dissected and exposed, and the kidney was mobilized to expose the tumor (Fig. 3C). After the renal artery was clamped with laparoscopic bulldog clamps (Fig. 3D), the renal parenchyma was resected with scissors ∼0.5 cm outside the tumor margin, and the tumor was removed (Fig. 3E).

The opened collection system was closed using 3-0 self-barbed sutures. The renal parenchyma was closed with 1-0 self-barbed sutures (Fig. 3F). Then, the bulldog clamps were released, and careful inspection was performed. The tumor was removed with a specimen bag, and a drainage tube was placed.

#### Transperitoneal approach

The patient was placed in a 60° contralateral decubitus position. A 1-cm incision was made at the midclavicular line of the affected side below the costal margin, and a pneumoperitoneum needle was inserted to inject CO2 gas until the pneumoperitoneum pressure reached 14 mmHg. A 12-mm port for the 30° 3D video laparoscope was placed supraumbilically at the outer edge of the rectus abdominis. Two 10-mm robotic cannulae were placed at the first puncture point and at the anterior axillary line below the umbilicus for the robotic instruments.

**FIG. 4.**
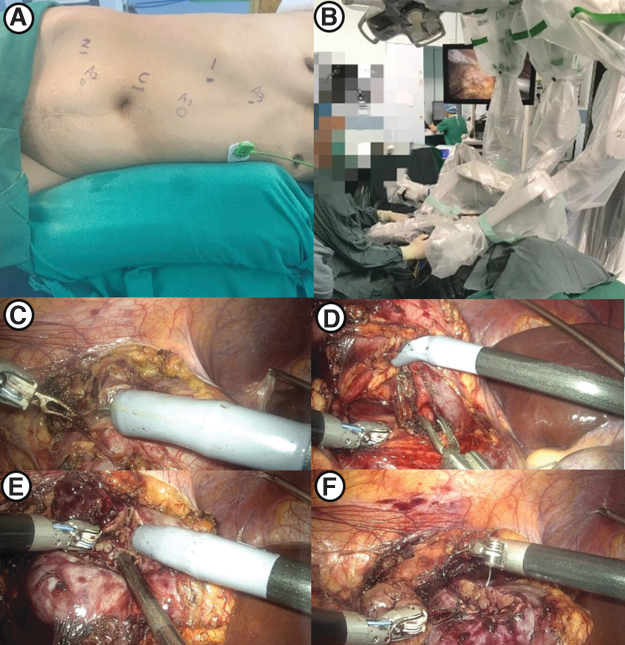
Surgical procedures of KD-SR-01-assisted PN using the transperitoneal approach. **(A)** Template of port placement using the transperitoneal approach (sites 1 and 2 for robotic instruments, site C for the camera, and sites A1, A2, and A3 for assistant ports). **(B)** Distribution of robotic arms. **(C)** Free kidney and exposed renal tumor. **(D)** Clamping of the renal artery. **(E)** Resection of the renal tumor. **(F)** Reconstruction of the renal parenchyma using continuous sutures.

Additionally, 12-mm and 5-mm assistant ports were placed on the midline 5 cm above the umbilicus and 5 cm below the umbilicus, respectively (Fig. 4A, B). Then, docking was performed. The colon was mobilized and pulled inside. The renal artery was dissected, and the kidney was freed to expose the tumor (Fig. 4C). After the artery was clamped (Fig. 4D), the renal parenchyma was resected ∼0.5 cm from the edge of the tumor and then the tumor was removed from the kidney (Fig. 4E).

The kidney wound was sutured in the same way as in the retroperitoneal approach (Fig. 4F). Then, the bulldog clamps were released. The tumor was retrieved with a specimen bag, and a drainage tube was placed.

### Study variables

The patient variables included age, sex, body mass index (BMI), and preoperative estimated glomerular filtration rate (eGFR); and the operative variables included robotic operative time (ROT), total operative time (TOT), warm ischemia time (WIT), estimated blood loss (EBL), and intraoperative transfusion. The postoperative variables included eGFR on the first postoperative day, eGFR on the fourth postoperative day, postoperative complications within the 30-day follow-up, length of hospital stay, histological characteristics, and surgical margin.

All patients underwent preoperative B-mode ultrasound and enhanced abdominal computed tomography/magnetic resonance imaging to confirm the diagnosis of renal tumors. Tumor complexity was assessed using the R.E.N.A.L. score^14^ and categorized as low (4–6) or moderate (7–9).

ROT was defined as the time from the beginning of the robotic surgical instrument-assisted operation to the removal of robotic surgical instruments. Docking time was defined as the time from moving the surgical system to the operating table until the last cannula was docked into the corresponding surgical arm. Complications were graded using the Clavien-Dindo classification system.^15^

### Evaluation

The main indicator was the efficiency of the operation, defined as meeting all of the following requirements: (1) the tumor was removed according to the established surgical plan without conversion to RN or open or conventional laparoscopic surgery and (2) the WIT was <30 minutes for patients with an R.E.N.A.L. score of 4–6 and <40 minutes for patients with an R.E.N.A.L. score of 7–9.

### Statistical analysis

The SPSS 23.0 statistical software program (SPSS, Inc., Chicago, IL) was used. The results are expressed as numbers (%) for categorical variables, as the mean ± standard deviation for normally distributed continuous variables, and as medians (ranges) for continuous variables with skewed distribution.

## Results

From December 2020 to March 2021, 17 patients (10 men and 7 women) underwent KD-RPN. The baseline characteristics of the study population are summarized in Table 1. The median age was 51 years (range, 36–72 years), and the median BMI was 25.9 kg/m^2^ (range, 20.9–32.9 kg/m^2^). The tumors were located in the left kidney in eight cases and the right kidney in nine cases. The median tumor was 3.3 cm (range, 1.4–4.0 cm). The R.E.N.A.L. score was 4–6 in 12 cases and 7–9 in 5 cases.

**Table 1.** Demographics, Tumor Characteristics, and Pathology

No. of patients	*n* = 17
Sex, male/female	10/7
Age (years), median (range)	51 (36–72)
BMI (kg/m2), median (range)	25.9 (20.9–32.9)
Side of tumor, left/right	8/9
Size of tumor (cm), median (range)	3.3 (1.4–4.0)
R.E.N.A.L. score, *n* (%)	
4–6	12 (70.6)
7–9	5 (29.4)
Histology, *n* (%)	
Clear cell	9 (52.9)
Papillary cell	2 (11.7)
Oncocytoma	1 (5.8)
Angiomyolipoma	5 (29.4)

BMI = body mass index.

The intraoperative and postoperative characteristics are summarized in Table 2. Four patients underwent transperitoneal procedures, while 13 patients underwent retroperitoneal procedures. KD-RPN was performed in all cases without conversion to RN or open or conventional laparoscopic surgery. The average TOT and ROT were 110.5 ± 37.6 minutes and 68.6 ± 26.0 minutes, respectively. The average docking time was 3.3 minutes (2.2–6.3 minutes). The median EBL was 50 mL (range, 50–200 mL). The average WIT was 16.9 ± 9.0 minutes.

**Table 2.** Intraoperative and Postoperative Characteristics

No. of patients	*n* = 17
Surgical approach, *n* (%)	
Transperitoneal	4 (23.5)
Retroperitoneal	13 (76.5)
Robotic operative time (minutes), mean ± SD	68.6 ± 26.0
Total operative time (minutes), mean ± SD	110.5 ± 37.6
Docking time (minutes), median (range)	3.3 (2.2–6.3)
Estimated blood loss (mL), median (range)	50 (50–200)
Transfusion, *n* (%)	0 (0)
Warm ischemia time (minutes), mean ± SD	16.9 ± 9.0
eGFR before the operation (mL/min), mean ± SD	97.9 ± 10.7
eGFR 1 day after the operation (mL/min), mean ± SD	91.7 ± 12.9 ^a^*p* = 0.036
eGFR 4 days after the operation (mL/min), mean ± SD	95.7 ± 13.4 ^b^*p* = 0.427
Intraoperative complications, *n* (%)	0 (0)
Postoperative complications, *n* (%)	
Clavien I	5 (29.4)
Clavien II–IV	0 (0)
Postoperative hospital stay (days), median (range)	5 (4–9)
Surgical margins, *n* (%)	
Positive margin	0 (0)
Negative margin	17 (100)

^a^ There is a reduction in the eGFR 1 day after the surgery compared with before the surgery.

^b^ There is no difference between the eGFR 4 days after the surgery and eGFR before the surgery.

eGFR = estimated glomerular filtration rate; SD = standard deviation.

There were no intraoperative complications. Postoperative complications were observed in five patients, requiring the need for antiemetic drugs in three cases and analgesics in two cases. No patients required blood transfusion perioperatively. The median time to discharge was 5 days (range, 4–9 days). Pathologic examination revealed clear cell carcinoma in nine patients (52.9%), papillary cell carcinoma in two patients (11.7%), oncocytoma in one patient (5.8%), and angiomyolipoma in five patients (29.4%). All surgical margins were found to be negative.

There was a significant decrease in eGFR on the first postoperative day compared with the preoperative eGFR (91.7 ± 12.9 mL/min *vs* 97.9 ± 10.7 mL/min, *p* = 0.036). However, no significant difference was observed between the preoperative eGFR and that on the fourth postoperative day (95.7 ± 13.4 mL/min *vs* 97.9 ± 10.7 mL/min, *p* = 0.427).

## Discussion

This is the first prospective study designed to evaluate the safety and feasibility of KD-RPN. In this study, all KD-RPN procedures were completed in accordance with the established plan without conversion to RN or open or traditional laparoscopic surgery. No intraoperative complications or major postoperative complications (Clavien grade >II) were observed. Our initial experience suggests that KD-RPN is technically feasible, valid, and safe.

In a previous study of a porcine model, the KD-SR-01 robotic system showed comparable results with 3D laparoscopic partial nephrectomy (3D-LPN) from an operative perspective, while KD-RPN had advantages over 3D-LPN from an ergonomic perspective.^13^ In another study, Fan and colleagues reported their initial experience with KD-SR-01 robotic pyeloplasty in 16 cases and confirmed its safety and feasibility.^16^ However, due to the need for fine dissection of renal vessels, complex reconstruction of the renal parenchyma, and a constrained WIT, PN is more challenging than pyeloplasty for both surgeons and the robotic equipment.

The KD-SR-01 robotic surgical system offers several unique features not possible in laparoscopic surgery, including a higher degree of freedom with the robotic instruments, motion scaling, and tremor reduction, which enable surgeons to control the surgical arms and instruments more precisely and synchronously in real time. In the present study, the average docking time was 3.3 minutes (2.2–6.3 minutes), which was shorter than that reported in previous literature.^17^

The following reasons may contribute to the result. First, the patient cart of KD-SR-01 is a liftable and rotatable suspended operating arm structure platform similar to da Vinci Xi. The movable patient cart can be placed anywhere around the operating table. It is equipped with a laser precise positioning function, which optimizes the docking process and significantly shortens the preoperative preparation time.

Second, the medical staff attended many training sessions and are experienced in animal experiments, which greatly shortened the learning curve. The average TOT and ROT were 110.5 ± 37.6 minutes and 68.6 ± 26.0 minutes, respectively. The average WIT was 16.9 ± 9.0 minutes, which is comparable with that in previous studies on the da Vinci system.^18,19^ In our experience, clear 3D visualization and good instrument maneuverability account for these results. A previous study reported that the learning curve for da Vinci-assisted PN was 16 cases in terms of TOT and 26 cases in terms of WIT for an experienced renal surgeon with extensive prior experience with LPN.^20^ Therefore, we believe that once a stable plateau has been reached, KD-RPN might show more favorable results in terms of operative time and WIT.

Robot-assisted partial nephrectomy can be performed with the transperitoneal or retroperitoneal approach, and each has its own advantages and drawbacks. The increased working space provided by the transperitoneal approach allowed adequate manipulation space for the devices, thus decreasing external robotic arm conflict. Conversely, the retroperitoneal approach is performed in the small space of the retroperitoneum cavity.

However, the retroperitoneal approach enables direct access to the renal artery without the need for mobilization of the colon, which might be advantageous for patients with prior abdominal surgery and for faster recovery of postoperative bowel function. A further potential benefit, which has already been described for the retroperitoneal approach, is that the retroperitoneal space may tamponade hemorrhage and prevent complications caused by the contact of urine with the peritoneum.^21–23^

In our series, KD-RPN procedures were mainly performed using the retroperitoneal approach. In our opinion, even in the small space of the retroperitoneum cavity, the KD-SR-01 system was competent for PN due to its high degree of freedom of the robotic instruments.

We have a preliminary understanding of both the advantages and disadvantages of the new platform. First, the open console allows for a natural neck posture, which helps relieve neck pain and stress. Second, the KD-SR-01 system can match the 3D laparoscopic display system of various brands that have been listed, which can greatly reduce the cost. Third, three surgical arms are suspended on the beam of a movable cart, which allows the arms to rotate as a group to adjust to the position of the patient. Fourth, the cart adopts force sensor technology, making it convenient for users to push it. Fifth, a cross-laser device is used to facilitate docking.

The lack of tactile feedback is the main drawback of the new platform. In the future, the new robot platform should be developed with a haptic feedback system. In recent years, robot-assisted single-site surgery achieved a better cosmetic effect, lesser pain, and faster recovery than traditional surgery.^24^ The da Vinci single-port (SP) surgical system has been available for several years, and preliminary data from centers of excellence show surgery with the SP system to be safe and feasible, with perioperative outcomes comparable with traditional multiport robotic surgery.^25^

However, high-quality comparative studies are currently lacking. In addition to the da Vinci SP system, other single-port platforms are under development and testing. Titan Medical (Toronto, Canada) focused on the SPORT Surgical System as a platform for robotic single-site surgery, which has an open console with 3D HD vision controlling a 3D flexible telescope with fiber-optic-based illumination and two flexible instruments.^9^ The insertable robotic effector platform was developed at Vanderbilt University, which consists of a 3D telescope and two flexible arms.^9^

Development of these new platforms will ideally promote innovation and drive the competitive price pressure downward for single-port technology. Furthermore, long-distance operations based on 5G technology will be the focus of future developments. Despite these shortcomings, the new robot platform might offer a new option for minimally invasive robotic surgery.

This study has some limitations. First, in the present study, the team performed 17 KD-RPN procedures, which might be insufficient to reach the stable plateau of the learning curve. Second, to explore the feasibility of KD-RPN, the patients were carefully selected and all candidates had renal tumors with an R.E.N.A.L. score less than 9. Further exploration is needed to assess suitability in more complex cases. Third, in the present study, the surgical team had extensive experience in performing robotic and laparoscopic PN. The learning curve of KD-RPN for surgeons lacking robotic and laparoscopic experience needs further exploration. Fourth, due to the short follow-up period, long-term oncologic and functional outcomes could not be assessed and should be further evaluated after a longer follow-up period.

## Conclusions

This is the first clinical assessment of the KD-SR-01 robotic surgical system for PN. Our initial experience demonstrated the feasibility and safety of KD-RPN. Despite the relatively small number of patients in our series, the advantages of the new robotic systems are difficult to ignore. Considering the current high costs of da Vinci robotic surgery systems, the emergence of new competitive devices will make robotic surgery more affordable.

We further emphasize that such investigations into the feasibility of robot-assisted procedures should initially be performed by surgeons who are experienced in both robotic and laparoscopic techniques to reduce patient morbidity during the learning phase.

## Abbreviations Used

3D: three-dimensional
3D-LPN: 3D laparoscopic partial nephrectomy
BMI: body mass index
CT: computed tomography
EBL: estimated blood loss
eGFR: estimated glomerular filtration rate
FDA: US Food and Drug Administration
KD-RPN: KD-SR-01 robotic partial nephrectomy
LPN: laparoscopic partial nephrectomy
MRI: magnetic resonance imaging
NMPA: National Medical Products Administration
PN: partial nephrectomy
PUMCH: Peking Union Medical College Hospital
RCC: renal-cell carcinoma
RN: radical nephrectomy
ROT: robotic operative time
SD: standard deviation
SP: single-port
SRM: small renal mass
TOT: total operative time
WIT: warm ischemia time
